# Effect of a trauma-informed care training program for a community care team working for young pregnant women: a longitudinal study

**DOI:** 10.1186/s12884-026-09241-8

**Published:** 2026-05-27

**Authors:** Junko Niimura, Miharu Nakanishi, Syudo Yamasaki, Satoshi Yamaguchi, Daniel Stanyon, Mitsuhiro Miyashita, Naomi Nakajima, Kaori Baba, Yuki Miyamoto, Masashi Kawano, Atsushi Nishida

**Affiliations:** 1 Mental Health Promotion Unit, Research Center for Social Science & Medicine, Tokyo Metropolitan Institute of Medical Science, 2-1-6 Kamikitazawa, Setagaya-Ku, Tokyo, 156-8506 Japan; 2 Department of Psychiatric Nursing, Graduate School of Medicine, Tohoku University, Miyagi, Japan; 3 Economic and Social Research Council (ESRC) Centre for Society and Mental Health, Institute of Psychiatry, Psychology and Neuroscience, King’s College London, London, UK; 4 Graduate School of Nursing Science, St. Luke’s International University, Tokyo, Japan; 5 Department of Psychiatric Nursing, Graduate School of Medicine, The University of Tokyo, Tokyo, Japan; 6 Mental Health Counseling, Private Practice, Tokyo, Ogikubo Japan

**Keywords:** Childhood trauma, Young pregnant women, Trauma-informed care, Training program, Program evaluation

## Abstract

**Background:**

Young maternal age (24 years or below) is a well-documented risk factor for child maltreatment. Young pregnant women are more likely than older mothers to have experienced childhood trauma, potentially increasing their vulnerability to re-traumatization during pregnancy and perinatal care interactions. Re-traumatization may play a role in increased child maltreatment risk, underscoring the importance of incorporating trauma-informed care (TIC) approaches into maternity services for this population. However, the implementation and evaluation of TIC training in community-based perinatal maternity care settings remain limited. As favorable staff attitudes are critical for the successful adoption and sustainability of TIC, evaluating the impact of training on professional attitudes is essential. We aimed to develop and evaluate the effects of a TIC training program on community-based maternity staff attitudes toward TIC in the Tokyo metropolitan area.

**Methods:**

Fifty-nine community-based maternity care staff (public health nurses, social workers, psychologists, and administrators) participated. The two-day training program was delivered in two formats: online (September 2021) and face-to-face (May 2022). Across the two days, the program included 2 h and 50 min of didactic lectures and 2 h and 30 min of interactive group discussion. Content covered core TIC principles, trauma-related responses, applications in perinatal care, and strategies to promote sustainable trauma-informed practice. Participants completed the 35-item Attitudes Related to TIC scale (ARTIC-35) at pre-training, post-training, and six-month follow-up. Mixed-effects modeling was used to examine changes in ARTIC-35 scores over time.

**Results:**

Participants were predominantly women (81.4%). The mean age was 42.5 years and most participants were public health nurses (42.4%), psychologists (25.4%), and social workers (23.7%). Mean ARTIC-35 scores increased significantly from pre-training (5.30, standard deviation = 0.55) to post-training (5.70, 0.59; adjusted coefficient β = 0.40, *p* < .001) and remained significantly higher at six-month follow-up (5.55, 0.66; β = 0.26, *p* < .001). Effect sizes were medium (Cohen’s *d* = 0.72) immediately post-training and small to moderate (0.47) at six months.

**Conclusions:**

The training program was associated with significant and sustained improvements in staff attitudes toward TIC. Future research should examine whether favorable attitudinal changes contribute to improving TIC practice and preventing child maltreatment.

## Background

Child maltreatment is a major global public health concern with long-term consequences for physical and mental health across the life course [1]. Children born to young mothers—aged 24 or below—are more likely to experience maltreatment than those born to older mothers [2]. This elevated risk is partly attributable to young mothers’ exposure to adversities, resulting in vulnerability to psychosocial stress and challenges in parenting. Women with a history of childhood adversity and trauma, including abuse, neglect, and household dysfunction, are more likely to become young mothers and face challenges such as prenatal depression, anxiety, and parenting struggles [3–5].

Women with histories of childhood trauma are also at increased risk of re-traumatization during pregnancy and the perinatal period [6]. This period involves frequent contact with health and social care professionals, which may expose women to trauma reminders. For young mothers in particular, vulnerability to re-traumatization may be intensified by developmental stage, age-related power imbalances, social stigma (e.g., assumptions of immaturity or parental inadequacy), and heightened surveillance within care systems, including repeated risk assessments and detailed questioning about parenting capacity [7–10]. In such contexts, routine perinatal services—such as physical examinations, home visits, and discussions about health behaviors or parenting—may inadvertently trigger trauma-related distress [8]. Re-traumatization can lead to anxiety, diminished trust in professionals, and avoidance of services as coping patterns for trauma [5, 11, 12]. Delayed or inconsistent engagement with antenatal care may, in turn, increase the risk of other maladaptive coping behaviors such as smoking or substance use and adverse birth outcomes, including preterm birth or low birth weight. Such persistent distress and service disengagement may result in poor maternal mental health, parenting quality, and child developmental outcomes, thereby reinforcing intergenerational cycles of disadvantage [13]. Given that the first few years of life are critical in lifelong health outcomes, sustained engagement with perinatal services is essential not only for strengthening parenting capacity and safeguarding child development but also for improving population health and health equity.

These risks underscore the need for trauma-informed care (TIC), an approach that seeks to prevent re-traumatization and service disengagement by fostering safe, respectful, and collaborative relationships and by enhancing professionals’ understanding of the impact of trauma [14, 15]. Recognizing age-related power dynamics and adopting a nonjudgmental stance are particularly important when working with young mothers [8]. Introducing TIC principles into community-based maternity services would be of particular importance in supporting young mothers at risk of experiencing socioeconomic disadvantage [9]. Community-based maternity staff belong to disciplines such as public health, maternal and child health, and child welfare; they are responsible for ongoing contact with mothers and their families during home visits and consultations from early pregnancy onward. However, despite growing recognition of the importance of TIC in perinatal care, the integration of TIC principles into training for community-based maternity staff remains limited [16–25]. Moreover, few studies have explicitly evaluated whether TIC training leads to measurable improvements in professionals’ attitudes. Shifts in professional attitudes precede sustainable changes in clinical practice and are considered foundational to the implementation and sustainability of TIC [26, 27].

To address the limited evidence base regarding TIC training in community-based perinatal settings and the need to evaluate attitudinal change among staff, this study examined the effectiveness of a TIC training program for community staff providing maternity care and home-visit services during the perinatal period. We hypothesized that participation in the training would be associated with improved attitudes toward TIC.

## Methods

### Study design

**Fig. 1:**
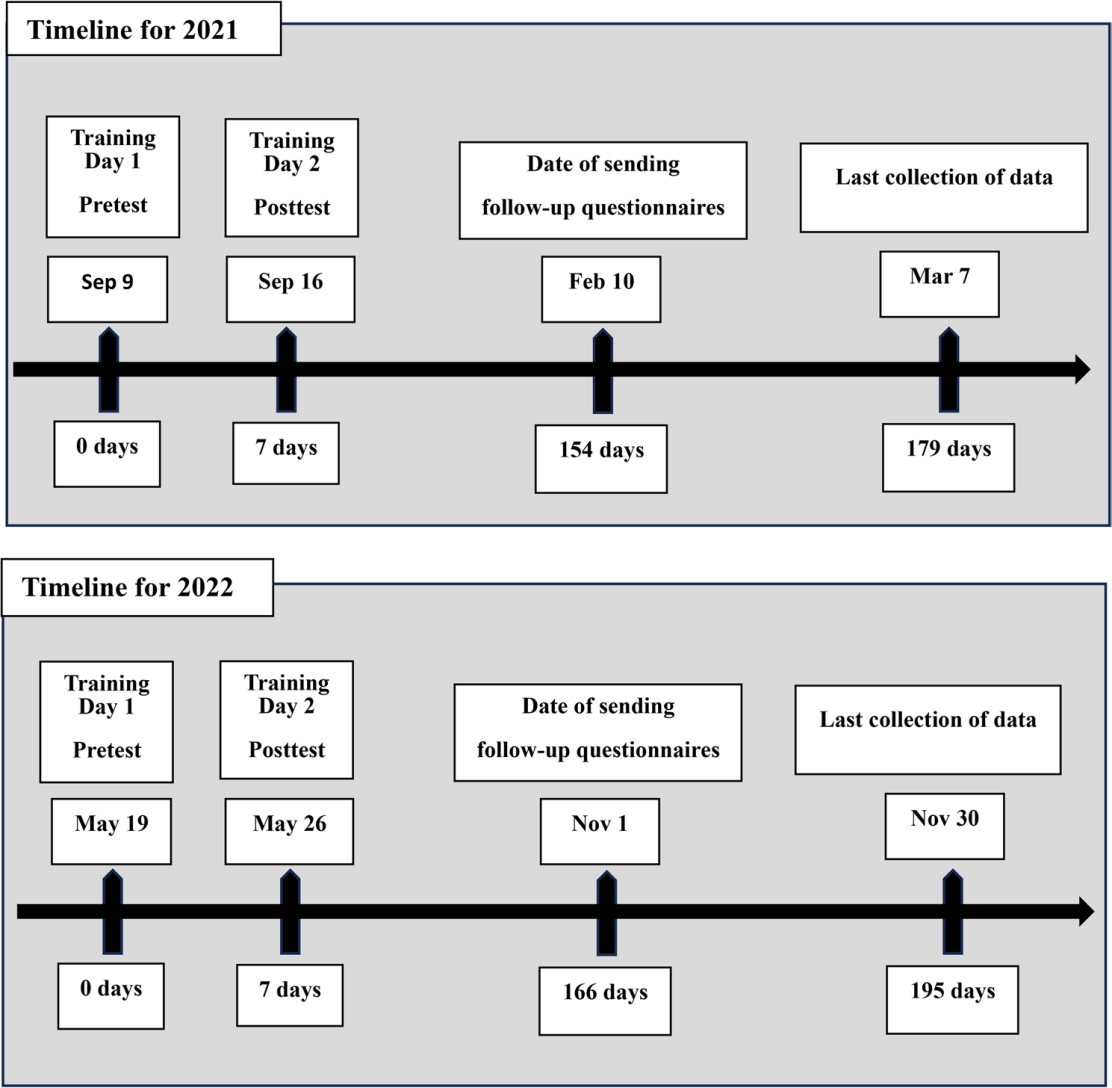
Study timeline

This study employed a pre–post design with a six-month follow-up assessment and was conducted at Institute A in Tokyo, Japan. Institute A is a public research institute established by the Tokyo Metropolitan Government that conducts applied and translational research in collaboration with municipalities and community service providers to improve health equity and service quality for Tokyo residents. In this study, the institute served as the coordinating center for program development, implementation, and evaluation.To recruit a sufficient number of participants, the training program was delivered to two cohorts. Group A participated between September 2021 and March 2022 and received the training online owing to COVID-19 restrictions. Group B participated between May and November 2022 and received the training face-to-face. A total of 61 community-based staff were enrolled in the study; 59 participants who completed the pre-training 35-item Attitudes Related to TIC scale (ARTIC-35) were included in the final analysis (Fig. 1). The study protocol was approved by the Ethics Review Board of the Tokyo Metropolitan Institute of Medical Science (Approval number: 21–44).

### Setting and participants

The study involved four municipalities in Tokyo that were preparing to implement a Tokyo Metropolitan Government policy initiative aimed at strengthening perinatal support for young women. In Japan, approximately 94.8% of pregnant women register their pregnancy at their local ward office during the first trimester to access publicly funded perinatal services provided by public health nurses [28, 29]. These services include individual interviews at pregnancy notification, home visits approximately one month after delivery, and routine infant health check-ups at four, six, and nine months. When risk of child maltreatment is identified, public health nurses collaborate with child welfare social workers and psychologists.

Each municipality appointed a research coordinator to recruit participants using purposive sampling. Eligible staff were qualified professionals (i.e., public health nurses, social workers, psychologists) or administrative personnel working in perinatal maternity care or home-visiting services. Coordinators were instructed to recruit both groups of professionals: frontline practitioners directly involved in supporting mothers and families and managerial staff responsible for program oversight. While an equal split was not required, efforts were made to ensure representation from both groups, including the recruitment of at least two managers per municipality where feasible. Eligible staff received written information about the study and training program, and those who provided written informed consent were enrolled.

### Training program

Perinatal home-visiting services play a critical role in supporting young pregnant women by addressing anxiety, fear, and social vulnerability related to past traumatic experiences [30]. However, young pregnant women with trauma histories may experience barriers to engagement, including anxiety, shame, and fear, underscoring the need for trauma-sensitive approaches [31, 32].

The TIC training program was designed to enhance community staff’s knowledge of trauma and TIC principles and to strengthen their understanding of how adverse childhood experiences and other traumatic events influence the emotions and behaviors of young pregnant women. The program aimed to support the practical application of TIC principles in routine perinatal maternity and home-visiting services.

The content (Table 1) was informed by existing TIC literature [14, 32–35]. Participants attended a two-day program structured to alternate between didactic lectures (10–45 min per session) and interactive groupwork sessions of comparable duration. Across the two days, the total training time was approximately 5 h and 20 min, including 2 h and 50 min of didactic lectures and 2 h and 30 min of interactive group discussion. The program was developed and led by MK, a professor of psychiatric nursing with expertise in TIC, who served as instructor and facilitator. The remaining authors (JN, YM, SY, NN, and KB) supported the program as co-facilitators during group discussions.

**Table 1:** Trauma-informed care training program content

Lecture including short group discussions (total lecture hours: 2.9 h; total groupwork hours: 2.5 h)	
First day	Main contents
1. Definition of trauma and TIC (lecture 10 min × 4, groupwork 10 min × 4)	・ Definitions of trauma and the rationale of TIC ・ Understanding the relationship between the 3 Es of trauma (Event(s), Experience of these events, and Effects)
2. Evidence of adverse childhood experiences (lecture 45 min, groupwork 15 min)	・ Understanding the prevalence and impact of childhood adversity experiences

Homework: Participants consider one specific example of TIC and non-TIC in their own workplace	
Second day	
3. Behavioral, social, and emotional responses to traumatic events (lecture 10 min × 4, groupwork 10 min × 4)	・ Understanding how traumatic events can affect a person’s behavior and emotions (post-traumatic stress disorder)
4. TIC and non-TIC in perinatal care (lecture 10 min, groupwork 15 min)	・ Using the homework assignment to discuss TIC and non-TIC for perinatal women in their workplace
5. Motivational interview and engagement with service users (lecture 30 min, groupwork 10 min)	・ Understanding essential perspectives and techniques for implementing TIC for perinatal women
6. Secondary trauma and compassion fatigue (lecture 10 min, group work 30 min)	・ Understanding the causes of burnout and caregiver fatigue and how to create an environment where TIC practices are sustainable
7. Closing	

*TIC* trauma-informed care

### Measures

Self-administered questionnaires were completed at pre-training, post-training, and six-month follow-up. Demographic information was collected using a study-specific questionnaire, which included age, gender, profession, years of professional experience, and prior experience with TIC training.

Attitudes toward TIC were measured using the ARTIC-35 [36]. Respondents rate items on a 7-point bipolar Likert scale reflecting their beliefs about their professional role during the previous two months. The ARTIC-35 comprises five subscales: (a) underlying causes of problem behavior and symptoms, (b) responses to problem behavior and symptoms, (c) on-the-job behavior, (d) self-efficacy at work, and (e) reactions to work. Higher total mean scores indicate more favorable attitudes toward TIC implementation.

The original ARTIC-35 demonstrated strong internal consistency (α = 0.91) [37]. We used the Japanese version of the ARTIC-35, which has been developed using a standard translation–back-translation process and has demonstrated validity and reliability [38]. In this study, Cronbach’s α coefficient was 0.89 at baseline, 0.93 at post-intervention, and 0.94 at six-month follow-up.

### Statistical analysis

First, we summarized the mean scores of the ARTIC-35 at pre-intervention, post-intervention, and six months after the program. Second, we examined changes in the ARTIC-35 scores to evaluate the effect of the program using a linear mixed-effects model. We adopted mixed models for analyzing longitudinal data to estimate correlations within data obtained from the same individual at multiple time points [39]. The models are robust against missing data in outcome variables under the assumption of missing at random, because their estimations are based on the maximum-likelihood method [40]. The equation for the model used in this study is as follows:

$$\:{\mathrm{Y}}_{\mathrm{t}\mathrm{i}}\:=\:{\mathrm{B}}_{0}\:+\:{\mathrm{B}}_{1}\:\left(\mathrm{p}\mathrm{o}\mathrm{s}\mathrm{t}\right)\:+\:{\mathrm{B}}_{2}\:\left(\mathrm{s}\mathrm{i}\mathrm{x}\:\mathrm{m}\mathrm{o}\mathrm{n}\mathrm{t}\mathrm{h}\mathrm{s}\right)\:+\:{\mathrm{e}}_{\mathrm{t}\mathrm{i}}\:+\:{\mathrm{u}}_{0\mathrm{i}}$$

where subscripts *t* and *i* refer to time and participant, respectively. The dependent variable (Y_ti_) is the ARTIC-35 score. The intercept and effects of the “post” and “six months” follow-up assessments are shown by B (unstandardized regression coefficients). These coefficients represent the effect of the program at each assessment timing (mean score difference between pre- and post-assessment [or follow-up assessment]). The residuals are shown by e_ti_. The assessment occasions (post and six months) were nested within the participant element; hence, the random effect (intercept) of participant (u_0i_) is included. We calculated effect sizes (Cohen’s *d*) from the mean score differences divided by the standard deviation (SD) of the ARTIC-35 score at pre-assessment [41]. According to Cohen’s conventional benchmarks [42], 0.2, 0.5, and 0.8 represent small, medium, and large effects, respectively. Our analysis included the data of 59 participants who completed the ARTIC-35 at pre-assessment. We set the level of significance at alpha = 0.05. In addition, a Monte-Carlo-based post-hoc power analysis was conducted. The analysis was performed using R version 4.1.3 with the lmerTest and simr packages.

## Results

### Participant demographics

Participant characteristics are presented in Table 2. Of the 61 community-based staff enrolled, 59 who completed the pre-training ARTIC-35 were included in the final analysis. Most participants were women (81.4%); their mean age was 42.5 years (SD = 11.3). “Public health nurse” was the most common job category (42.4%), and the participants had a mean professional experience of 12.4 years (SD = 10.4). In addition, 30.5% of the participants, including 70.2% of psychologists, 16.7% of social workers, and 11.1% of public health nurses, had received previous TIC training for non-pregnant women conducted by other organizations.

**Table 2:** Participants’ demographics (*N* = 59)

Demographic characteristics	*n*	%
Women (*n* = 58)	48	81.4
Employment position (*n* = 59)		
Managerial	16	27.1
Staff	38	64.4
Other	5	8.5
Job category (*n* = 59)		
Social worker	14	23.7
Public health nurse	25	42.4
Psychologist	15	25.4
Clerk	3	5.1
Other	2	3.4
Education (*n* = 58)		
High school	1	1.7
Vocational school/Junior college	14	23.7
University	29	49.2
Graduate school	14	23.7
Experience of prior trauma-informed care training (*n* = 59)	18	30.5
Age in years, *M* (*SD*) (*n* = 59)		42.5 (11.3)
Years in job role, *M* (*SD*) (*n* = 59)		12.4 (10.4)

*M* mean, *SD* standard deviation

### Intervention effects

Table 3 presents the results of the linear mixed-effects model examining changes in ARTIC-35 scores over time. At pre-training, the mean ARTIC-35 score was 5.30 (SD = 0.55). The mixed-effects model indicated that ARTIC-35 scores were significantly higher at post-training compared with pre-training (β = 0.40, 95% confidence interval: 0.27–0.52, *p* < .001). This increase represents an improvement of approximately 7.5% relative to the pre-training mean score. At the six-month follow-up, scores remained significantly higher than at baseline (β = 0.26, 95% confidence interval: 0.14–0.38, *p* < .001), corresponding to an improvement of approximately 4.9% relative to the pre-training mean. Effect sizes were medium (Cohen’s *d* = 0.72) at post-training and small to moderate (0.47) at six months. Random-effects estimates indicated variance components of 0.105 for time (eti) and 0.259 for participants (u0i). Based on Monte Carlo simulation, the estimated statistical power exceeded 0.99 for the observed effect sizes with the current sample size (*n* = 59).

**Table 3:** Intervention effects at pre-intervention, post-intervention, and six-month follow-up

	M (SD)	
Pre	5.30 (0.55)	
Post	5.70 (0.59)	
6 months	5.55 (0.66)	
Fixed effects:	Regression coefficients (95% confidence intervals)	Effect size
Intercept (Pre)	5.30*** (5.14, 5.45)	—
Post (Reference: Pre)	0.40*** (0.27, 0.52)	*d* = 0.72
6 months (Reference: Pre)	0.26*** (0.14, 0.38)	*d* = 0.47
Random effects:	Variance	
Time (e_ti_)	0.105	
Participants (u_0i_)	0.259	

*M* mean, *SD* standard deviation

***p < .001

## Discussion

We evaluated the effect of a TIC training program on attitudes toward trauma-informed practice among community maternity care staff using the ARTIC-35. Participants demonstrated significant improvements in attitudes toward TIC, with effects maintained at six-month follow-up.

These findings are consistent with previous studies reporting improvements in professional attitudes following TIC training in perinatal care [17], pediatric care [25], residential treatment settings [19], educational contexts [18], and among other community stakeholders [22, 23]. Our study extends this literature by adapting TIC principles into training for community-based maternity care staff, who are vital to empowering young pregnant women. Given the heightened psychosocial vulnerability, developmental stage, and structural dependency associated with young mothers, strengthening trauma-informed approaches within services targeting this population is particularly important [43].

One possible explanation for the observed improvement in attitudes relates to the program’s structure, combining brief didactic sessions with facilitated small-group discussions. This format encouraged participants to reflect on and translate their learning into daily practice [44, 45]. However, nearly one-third of our participants had received TIC training from other organizations prior to our program; thus, their readiness was potentially already at a higher level, which is a prerequisite for more favorable attitudes toward TIC practice. Future studies should examine whether the level of intervention effects on attitudinal changes varies by baseline training status.

Improvements in attitudes were retained at six-month follow-up. One possible explanation is the inclusion of both frontline and managerial staff in the training. Shared participation may have facilitated a common understanding of TIC principles and supported organizational reinforcement of trauma-informed perspectives over time. Organizational commitment is widely recognized as a critical factor in embedding TIC into routine practice [46–48]. However, we acknowledge that involving management-level staff in multi-hour training sessions may not be feasible in all public health settings. Future research should explore scalable and time-efficient adaptations to enhance feasibility and dissemination.

This study has several limitations. Although post hoc power analysis indicated adequate statistical power to detect the observed within-sample changes, the relatively small sample size limits precision and generalizability. The absence of a control group further limits causal inference. A randomized controlled trial design is needed to determine the independent effect of the training. Furthermore, we assessed only professional attitudes toward TIC. Future research should examine whether improvements in attitudes translate into measurable changes in service delivery and service user outcomes, such as engagement with perinatal services, perceived safety, or satisfaction with care.

## Conclusion

To our knowledge, this is the first evaluation study on TIC training among community staff providing maternity care and home-visit services in the perinatal period using a standardized measure of professional attitudes. Participants indicated meaningful and sustained improvements in attitudes toward trauma-informed practice for young pregnant women. Our program may contribute to workforce development with TIC principles for community-based maternity care staff. Further investigation is needed to determine if favorable attitudinal changes in turn improve TIC practice and prevent child maltreatment among young mothers.

## Data Availability

The datasets generated during and/or analyzed in the current study are available from the corresponding author on reasonable request.

## Abbreviations

ARTIC-35: Attitudes Related to Trauma-Informed Care
SD: Standard deviation
TIC: Trauma-informed care
